# Hospital and Institutional News

**Published:** 1916-08-19

**Authors:** 


### HOSPITAL AND INSTITUTIONAL NEWS.

### WHISTLING FOR "TAXIS" IN LONDON.

As a result of many letters to the Press upon the suffering occasioned to wounded soldiers and other residents of London, both sick and well, by the incessant whistling for taxi-cabs late at night which many selfish and thoughtless persons indulge in, it does seem, from the Home Secretary's statement in the House on Tuesday that he is proposing a regulation to take effect on Monday, August 21, prohibiting the use of cab-whistles during the night in a certain specified Metropolitan area or areas, as if at last this abominable nuisance may be mitigated. It is true, no doubt, that diminished numbers of cabs on the streets have led to an increase in the amount of fruitless whistling; and that the complaints of wounded soldiers have received more attention than those of sick civilians before the war. But the whistling nuisance has been a disgrace to London for some years, and should have been put down long before the war. The writer recalls his sentiments towards some thoughtless neighbours who aroused him from slumber at midnight some four or five years ago by whistling for a cab: he counted sixty-three separate blasts, after which the departing guests were heard to remark that as it was a fine night they would walk home, the distance being so short! Londoners have got into this bad habit owing to slackness on the part of the authorities, and it will probably require drastic action in two or three cases before they will give it up.

### A NEW AUSTRALIAN WAR HOSPITAL.

The Imperial authorities have accepted the offer made by the Commonwealth Ministry of a new general hospital for service with the Australian Imperial Forces. It will be known as the 14th Australian General Hospital, and will contain 1,040 beds. It is stated in the Melbourne papers that one lieutenant-colonel, eight majors, and ten other medical officers are to be nominated in Australia: the commanding officer and the senior surgeon are to be selected from amongst officers now serving with the Forces. This makes a total of twenty-one medical officers, exclusive of two quartermasters, a dentist, a pharmacist, and a masseur. Such a large medical staff is unnecessary and wasteful. Most of the hospitals in the British Expeditionary Force of equal accommodation have been, during the last twelve months, run with from eight to twelve medical officers; and although it is true that at times of strain they have been somewhat understaffed, we should regard fifteen or sixteen officers as amply sufficient for No. 14 Australian General Hospital. Nor do we quite see why two quartermasters should be required. The large proportion of majors is also an unnecessary extravagance. Seventy-nine nurses will be required. Presumably about half to two-thirds will not be fully trained; for seventy-nine nurses with full hospital training would be a needlessly large number.

### AN EXCLUSIVE SUBURB AND DISABLED SOLDIERS.

Considerable excitement has been aroused in Liverpool over the refusal by the War Pensions Statutory Committee of Mr. W. E. Cain's mansion, Wilton Grange, West Kirby, as a home for permanently disabled soldiers. Indeed, there are signs that this refusal is being reconsidered, and speculation has been rife as to the reasons on which it was based. It is suggested that, until the recent decision of the Government to grant six millions to the Statutory Committee, shortage of funds might have been one. But it is also tentatively hinted that local objections were made to the proposal. These, in spite of much hedging, seem to resolve themselves into the feeling on the part of certain residents that the exclusiveness of their suburb, or the value of their property, might be infringed or affected if a home for disabled soldiers were established in their midst. Mr. Cain is reported to have declared that it was suggested to him that this grave objection would vanish if Wilton Grange were used for officers only! As the matter is of public importance the objectors will have to put forward their reasons. The property cost £25,000, and public opinion in Liverpool and West Kirby is not likely to allow so handsome an offer to be refused without knowing quite plainly the reasons for such a decision.

### SHOULD THE CHURCHES BE CONVERTED INTO HOSPITALS?

The transformation of St. John's Presbyterian Church, Northwood, into a hospital ward is an event in the history of adapted buildings which deserves to be recorded at this time. It follows the use of the lecture-hall attached to it, which became the local V.A.D. Hospital three months after war was declared. The demand for 100 instead of 50 beds, consequent upon the losses entailed by the offensive in the West, induced the minister, officials, and congregation to offer their church in the place of their lecture-hall, where the services are now being held. At a time when a raid is being made upon the schools for hospital purposes the example of the first church in the country in offering itself for the accommodation of the wounded is a noteworthy precedent, which force of circumstances has made a frequent occurrence in France. The Chaplain-General to the Forces, Bishop Taylor Smith, is reported to have warmly commended the scheme. Will it be generally followed? The clergy and congregations who use their churches only once a week should face the question. No one, so far as we know, has ventured to criticise the action of the Presbyterians at Northwood.

### CLUBS AND HOTELS AS HOSPITALS.

The raid on the schools for hospital purposes which is accompanying the enthusiastic cry for the reform of our educational system is a stroke of irony which seems to show the scepticism of the public concerning its most loudly trumpeted beliefs. The general unsuitability of school buildings for conversion into hospitals has been often insisted on in these columns, and Mr. Frank Briant, of the London County Council, has been following up our suggestion that hotels are a type of building far more suited to adaptation, by urging that not only hotels but clubs should be employed before a further finger is laid upon the schools, already so extensively commandeered. The public health aspect of the question also must not be lost sight of. Mr. Briant well asks what is to become of the displaced children, and points out that they must either be pressed into already crowded buildings, or left by the half-sessions system in the streets for a large part of the day. He adds that children under five years of age are being refused admission because no accommodation can be found for them, and pertinently asks whether it will seem patriotic to the men in the trenches to know that their children are being hustled into crowded buildings or left in the streets. As we note elsewhere, one church has offered itself; and the adaptation of suitable churches, hotels, and clubs is less open to objection than that of schools.

### A JOKE IN A WILL.

WILLS usually provoke more heart-searchings than laughter, and the tradition of bitterness extends to the following somewhat acid joke which accompanies a bequest by the late Mr. Samuel Rapkin to "Bart's." and certain other London hospitals. The testator, who was a fish salesman of Billingsgate Market, and left £12,682, after certain charitable bequests, stated that "as certain of my children are Liberal in politics they shall be liberal by nature." So before a share of the residue is paid to them they are to pay £200 to St. Bartholomew's Hospital and £100 each to the London, the West Ham, the Poplar, and the Woodford Jubilee Hospitals. The testator shows serious confusion of mind in this little *jeu d'esprit*, for by its terms he makes his children not liberal by nature, but in spite of themselves. Attempts to "reform" people by force are deservedly futile.

### THE EAST SUSSEX HOSPITAL: A BIG MISCALCULATION.

THE new buildings committee of the East Sussex Hospital, Hastings, is continuing with the work of the foundations of the new hospital. It was recently stated, however, by Major W. H. Mullens that the cost for the foundations of the new hospital had been under-estimated by £6,000. This apparently involves a serious error on the part of the new buildings committee which will not increase public confidence in its deliberations. Perhaps Major Mullens will explain how it occurred.

### GLOUCESTER RED CROSS HOSPITAL'S NEW WING.

BY reason of the important additions now completed, the Gloucester Red Cross Hospital becomes, we believe, the largest institution of its kind in the Gloucestershire area. It can now accommodate 200 men, or at a pinch even 240, thanks to the addition of the new Kitchener Ward, with the new dining-room, that are included in the new wing of the institution. The ward, constructed of wood, with a roof double-felted and distempe[illegible] and measuring 124 feet by 24 feet, accommo[illegible]es fifty or sixty beds, and has an electric installa[illegible]on of its own and its own system of drainage. The dining-hall, which is constructed in sections, seats 150 at four tables. The new building is noteworthy from the fact that the new ward has been carried out within three weeks of its conception and the dining-room within one week more. The cost has been defrayed by private donors who have remained anonymous.

### THE HAROLD FINK HOSPITAL.

IT is announced that Mrs. Catherine Fink has asked Mr. Dougl[illegible]ields to administer a fund of £5,000 with which she has end[illegible]ed beds at the Harold Fink Memorial Hospit[illegible]7 Park Lane, for the free treatment of office[illegible]isabled in the war. The Duchess of Portland, it is also stated, will nominate patients for these beds. Mr. Shields is an Australian surgeon who has for some years managed a very luxurious and expensive nursing home at the address in question. Since the war a large part of this home has been devoted to the treatment of wounded officers; Sir Alfred Fripp, we believe, and Mr. Shields himself have been in medical charge. We are not quite clear whether the announcement of Mrs. Fink's generosity indicates a change in the general scope and character of the work, nor whether the relief of disabled officers there is to continue after the war is over. The name Harold Fink Memorial Hospital possibly implies that the £5,000 now announced is only a part of Mrs. Fink's benefactions to No. 17 Park Lane.

### THE TREATMENT OF CAMP WORKERS.

A REQUEST has been made to the War Office for a grant towards the cost incurred by the Guardians in the treatment of camp workers at the Ripon Union Infirmary. In dealing with the official argument that the camp had been of great financial benefit to Ripon, it was pointed out that the infirmary was maintained not only by the ratepayers of the city, but of the rural district and a part of the North Riding. In the country districts it was declared that the presence of the camp had brought about adversity and actual financial loss, and therefore that to ask the ratepayers of these districts to bear the heavily increased expenditure to which the Union Infirmary had been put was unfair. All cases of serious illness among camp workers are stated to be brought to the infirmary. Is it not the duty of the War Office to be responsible for their treatment?

### THE WOUNDED AT ESSEX COUNTY HOSPITAL.

THE chief event of the past few months in connection with the Essex County Hospital has been the reception of an increased number of wounded and the change in the accounts consequent thereon.

At present the hospital accommodates 190 wounded, and, in spite of the fact that the usual sources of income have remained fairly constant, though donations have shrunk, the half-yearly income of the institution has risen to £4,635, as against £2,307 for the corresponding period of last year. The principal item in this development is £2,772 received from the War Office, which last year contributed £31. Of course, the expenditure has considerably increased also, and stands at £5,418 against £3,370, so that there is still a balance on the wrong side. Mr. H. B. Dickinson, vice-chairma[illegible] of the hospital committee, lately declared that far from this influx of soldiers having adversely affected the sick civilian population, the conditions of the hospital had been improved. It would be interesting to know how this gratifying result has been attained, and we hope that public gratitude may express itself in a revival of income under the heading of donations, which need to recover from their recent decline.

### T[illegible] HULL SANATORIUM.

The Hull Co[illegible]ration's new sanatorium on the Cottingham Castle estate has now been opened. The estate covers 100 acres, of which 33 have been devoted to the institution's buildings and grounds, while the rest of the land is to be used as the site for an isolation hospital. The sanatorium, which is on the pavilion plan (and includes a block of forty beds for advanced cases), contains eighty beds, and has cost £25,238. It is the boast of the Health Committee, of which Mr. F. Askew is chairman, that the local authorities of Hull were one of the first to submit proposals to the Local Government Board on the basis of the Astor Report. The Government grant has amounted to £10,800. It will be recalled that the foundation-stone of the sanatorium was laid by electricity by the King two years ago.

### SKETCHES IN HOSPITAL.

The chief event recorded in the August number of the *Gazette* of the 3rd London General Hospital, Wandsworth, is the departure of the commanding officer, who is taking out to the East a 1,040-bed hospital officered by Territorial medical officers. He takes with him between thirty and forty non-commissioned officers and men. The new C.O. is Sir Alfred Pearce Gould, whose connection with the 3rd London has been previously noted in the *Gazette*. Apart from its work for the patients and the distinguished artistic reputation of the staff, the hospital has been one of the most widely advertised of its class, the doings of which have frequently figured in the newspapers. There is an entertaining article by Miss H. M. Nightingale (a regular contributor) which might be entitled "How I Began," and some capital sketches by Private G. H. Dowd and Private G. A. Grant; and the way in which the life of the hospital (Sketches in the Main Hall: *en route* to the Concert, and to the Theatre, by Miss G. C. Ryle and Miss Marjory C. Collins) has been made use of as material for the illustrations and articles is especially commendable.

### COMPULSORY ANÆSTHESIA IN FRANCE.

A Special Commission has been appointed in Paris to consider the question as to whether soldiers should continue to have the right to refuse to be placed under anæsthetics. The right of a soldier to refuse a cutting operation is recognised, in France, as absolute from a legal point of view, because every such operation implies a certain risk. Also the soldier has the right to refuse simple anæsthesia without operation; and, according to the Paris correspondent of the official organ of the American Medical Association, Dr. Reynier has put forward the proposal to render compulsory the anæsthetisation of soldiers whenever the physician treating the case shall judge it necessary for the purpose of diagnosis or of treatment. He argues that so long as the soldier has the right to refuse to submit to anæsthesia it is often impossible to detect malingerers or to demonstrate the hysterical nature of certain contractures, and that consequently it is sometimes necessary to discharge men who might well be cured and are fit to take their place in the ranks. There is some opposition to Dr. Reynier's view on the ground that post-chloroformic sequelæ are by no means negligible, and that therefore the wishes of the patient ought not to be ignored: this suggestion is baseless, in our opinion.

### SEND ONE CHILD TO THE COUNTRY.

It is sad to find that the Children's Country Holidays Fund, 18 Buckingham Street, Strand, is short of money, and that during these summer days which have come at last it is not possible to send the children, as they ought to be sent, from the close courts and back streets of the town to the country. The fathers of the children are our fighting men and our munition workers, and the mothers, too, are loyally doing their duty. We ask each reader, Have you sent one child to the country, or, better still, are you sending one every week on a system? If not, please wake up and, whoever you are, man or woman, join hands with the Children's Country Holidays Fund to-day. Fourteen shillings keeps one child in the country for a fortnight, and no child is sent to the country by this Fund for less than a fortnight.

### FIFTY MILLIONS IN WAR GIFTS.

In the last number but one of *Land and Water*, Mr. W. E. Dowding has given some interesting tables of statistics and computations concerning the war gifts and charities of the British people. Somewhat unexpectedly, he finds that funds for the relief of distress and for the re-establishment of men returning to civil life exceed the moneys subscribed for the sick and wounded. The Prince of Wales' Fund aggregates six millions, and local funds for similar (but local) purposes are estimated at three millions more. Grants from employers, regular contributions from wage-earners in bulk, and similar relief to wives and families from communities of people, as opposed to public funds, are reckoned at ten and a half million, though this is admittedly a rather hazardous approximation. Adding a million for the Soldiers' and Sailors' Families Association and two kindred organisations, Mr. Dowding arrives at a total of twenty millions for the relief of distress. The *Times* Fund and the Scottish and Irish branches of Red Cross work account for five and a half millions; and counting in a vast volume of unpaid labour and other contributions in kind, six millions seems a conservative estimate. An equal sum is believed to have been given in "comforts" of many different kinds for the sick and wounded; and another million is put down for the cost of their entertainment. Ten millions have been spent upon the relief of our Allies, chiefly on the Belgians. Five millions raised overseas and two millions miscellaneous completes a total of fifty millions sterling, all raised by voluntary efforts.

### INCREASING DUTIES OF MEDICAL OFFICERS OF HEALTH.

It looks as though medical officers of health, more particularly those in the smaller boroughs and urban and rural areas, will have yet another duty thrust upon them in the near future. They are already working at war pressure, and in many instances have shouldered responsibilities which are outside the real scope of their position. Now it appears that they may be called upon to act as sanitary inspectors and inspectors of nuisances in the place of men who have been called up. At Forfar the medical officer is at the moment discharging temporarily the duties belonging to the sanitary officer, and a similar position has been brought about at Monmouth owing to the Gower Tribunal's refusal to exempt the nuisance inspector. Presumably public health authorities who thus add to their medical officer's work and responsibility will be asked for a corresponding addition to his salary. As regards the question of efficiency, there are undoubtedly very many medical officers of health who in peace-time work very light hours and can well be asked to do more during war; but this certainly does not apply to all, though perhaps of the majority it is accurate enough.

### SHOULD SATURDAY FUND SUBSCRIPTIONS BE RAISED?

The success attending the Hospital Saturday Fund in Birmingham is leading to a consideration of the best way to maintain the present income after the end of the war, when fewer people would be employed in the city. The prospect is serious from the fact that the fund has extended its already considerable convalescent work by opening a new home for twenty-four women at Malvern, and hastening the completion of a scheme for one at Droitwich for men and women suffering from rheumatism. It is suggested that individual contributions might be increased, and as the large majority of women contributors subscribe ½d. a week, while the men subscribe 1d., the women are to be asked if they can raise the larger sum. It is also pointed out that about one-third more beds are provided for women, so that the latter are more costly to the fund. The sum of 1d. a week all round, it is thought, would be sufficient to maintain the present total. Moreover, as a married man's penny provides benefits for his wife, the committee are asking if the married man will subscribe for his wife and increase his subscription to 1½d. or 2d. a week. The delegates have been instructed to bring the matter before the workpeople and to report their feeling on the matter to the committee. It is simply a question as to whether or not the subscribers wish the extra work to continue after the war.

### THE CASE FOR BUTTER IN PHTHISICAL WARDS.

A lively debate took place at a recent meeting of the Keighley Board of Guardians with reference to the giving of margarine to phthisical patients, who had made strong complaints on the subject. The Chairman, Mr. G. W. Pearson, pointed out that the dietary table was a matter for the doctor, but he would allow the motion put forward by one member that the doctor be recommended to substitute butter for margarine. After strong statements had been made as to the impropriety of giving margarine to people suffering from consumption, the motion was agreed to unanimously. We have often pointed out that, analytically considered, a high-standard sample of margarine apparently contains as much nutriment as butter, but there are two objections against its use in cases of illness which have to be considered. The first is that there is no fixed standard of quality, so that one kind of margarine may be vastly inferior to another. The second is that even the best margarine is not nearly so palatable as butter, and palatability plays an important part in digestion, especially in the case of those suffering from wasting diseases, whose strength requires to be built up by a generous diet. As the argument in favour of margarine is at bottom an economic one, we do not think in this case that the doctor need resent a technical interference in the dietary on the part of the board. If the board be prepared to pay the additional cost of butter, let the phthisical patients have it by all means.

### THIS WEEK'S DRUG MARKET.

The downward tendency of prices generally is still noticeable, although the actual reduction in quotations from week to week is small. In bromides there is little change, but there is a feeling that any further substantial reduction in prices is not probable. Japanese camphor is again much dearer, and it is not unlikely that English refined may advance in sympathy. Tartaric acid and citric acid are again somewhat cheaper, and cream of tartar still has a downward price tendency. Acetanilide is again lower. Acetylsalicylic acid is also rather lower. Almond oil is slightly cheaper. There is no quotable change in salicylates, but in face of the downward tendency of prices buyers are acting with caution. Pilocarpine is rather dearer. Phenolphthalein is unobtainable.

